# What if it is the eyes? The effect of eye movement disorders on cognitive testing in MS

**DOI:** 10.1007/s00415-026-13905-y

**Published:** 2026-06-02

**Authors:** S. N. Hof, E. Keytsman, T. A. N. Fuchs, M. H. J. Wessels, D. J. de Jong, E. M. M. Strijbis, M. M. Schoonheim, L. J. van Rijn, A. Petzold, B. M. J. Uitdehaag, J. Van Schependom, B. W. van Oosten

**Affiliations:** 1 MS Center and Neuro-ophthalmology Expertise Center Amsterdam, Amsterdam UMC location Vrije Universiteit Amsterdam, Neurology, De Boelelaan 1117, Amsterdam, The Netherlands; 2 Amsterdam Neuroscience, Neuroinfection and inflammation, Amsterdam, The Netherlands; 3 AIMS Laboratory, Centre for Neurosciences, Vrije Universiteit Brussel, 1050 Brussels, Belgium; 4 MS Center Amsterdam, Amsterdam UMC location Vrije Universiteit Amsterdam, Anatomy and Neurosciences, De Boelelaan 1117, Amsterdam, The Netherlands; 5 Neuro-ophthalmology Expertise Center Amsterdam, Amsterdam UMC location Vrije Universiteit Amsterdam, Ophthalmology, De Boelelaan 1117, Amsterdam, The Netherlands; 6 Onze Lieve Vrouwe Gasthuis, Ophthalmology, Amsterdam, The Netherlands; 7 The National Hospital for Neurology and Neurosurgery, Moorfields Eye Hospital and the Queen Square Institute of Neurology, UCL, London, United Kingdom; 8 Department of Electronics and Informatics (ETRO), Vrije Universiteit Brussel, 1050 Brussels, Belgium

**Keywords:** Internuclear ophthalmoplegia, Cognitive impairment, Symbol digit modalities test, Oculography, Eye tracking

## Abstract

**Background:**

Visual cognitive tests that rely on saccades may be affected by internuclear ophthalmoplegia (INO), irrespective of cognitive ability. INO and cognitive decline are common in people with multiple sclerosis (PwMS). We assessed the impact of INO on visual versus non-visual cognitive tests in PwMS.

**Methods:**

PwMS (*n*=197) completed auditory and visual neuropsychological tests, and demographically corrected *Z*-scores were obtained. INO was quantified using the DEMoNS infrared oculography protocol, applying the versional dysconjugacy index (VDI) of area under the saccadic trajectory (AUC) and peak velocity amplitude ratio (pV/Am). A subset (*n*=102) was reassessed after 6 years. Regression models were adjusted for disease duration, disability, cortical grey matter, and thalamic volume.

**Results:**

The presence of (mild) INO alone (*n*=66, 34%) was not associated with visual cognitive test performance after adjusting for disease characteristics. However, more severe right-sided INO on leftward gaze was associated with poorer performance on the Symbol Digit Modalities Test (SDMT) and Concept Shifting Test (CST): 0.1 increase in leftward VDI-pV/Am corresponded to SDMT and CST *Z*-score reductions of −0.08 (95% CI −0.13 to −0.03) and −0.14 (95% CI −0.22 to −0.07), respectively. This effect was not observed for the Paced Auditory Serial Addition Test (PASAT). Longitudinally, new right-sided INO (*n*=2) corresponded with CST performance decline (*Z*-score −3.96, 95% CI −5.88 to −2.05), but not after adjustment for disease characteristics.

**Conclusions:**

Severe right-sided INO impairs performance on cognitive tests requiring rapid eye movements (e.g. SDMT, CST), independent of cognitive ability. These tests should be interpreted with caution in people with severe INO.

**Supplementary Information:**

The online version contains supplementary material available at 10.1007/s00415-026-13905-y.

## Introduction

Multiple sclerosis (MS) is the most common chronic, immune-mediated, demyelinating disease of the central nervous system [41]. MS can affect many neurological functions, including oculomotor and cognitive function. Cognitive impairment affects 34–65% of people with MS, rising to as high as 80–90% in progressive disease [5, 8, 46]. It is gaining recognition as an independent symptom of MS and is a major determinant of quality of life [9, 27]. The evaluation of cognitive deficits in MS relies on neuropsychological testing, with the Symbol Digit Modalities Test (SDMT) for information processing speed emerging as the most widely used test due to its brevity, high reliability, and psychometric validity [6, 14, 35]. As a result, the SDMT is the recommended cognitive outcome measure for clinical trials and is often one of the few cognitive outcomes included [5, 21].

However, the SDMT not only assesses information processing speed but also requires abilities such as working memory and linked learning, and depends on sufficient visual, oral–motor and oculomotor functions [6, 10]. Therefore, performance may be affected by visual disorders and, critically, by eye movement dysfunction. Internuclear ophthalmoplegia (INO) is a common eye movement disorder in MS that can be observed in 25–34% of all persons with MS (PwMS) [18, 25]. In MS, INO results from demyelination and axonal damage to the medial longitudinal fasciculus, which coordinates synchronous horizontal eye movements. This leads to impaired horizontal eye movements with slowed or limited adduction of the ipsilateral eye and, optionally, an abduction nystagmus of the contralateral eye [3, 23, 25]. Patients with INO may experience diplopia, oscillopsia, reading fatigue, visual confusion, and loss of stereopsis [18]. Infrared oculography now provides an objective and accurate method to detect INO [15, 17, 25, 26].

Previous research has shown that PwMS with INO perform worse on the SDMT, but it remains unclear whether this difference in performance is attributable specifically to impaired eye movements rather than underlying cognitive dysfunction [18]. Moreover, the impact of INO and impaired saccades may be broader than the SDMT. During reading a rapid ‘return-sweep’ saccade in required to find a line. As such, any test that requires reading of multiple lines can potentially be affected by impaired saccades. There are other cognitive tests that require rapid eye movements, such as the Concept Shifting Test, for which the impact of eye movement disorders on test performance is unknown [39].

Given the high prevalence of both cognitive impairment and INO in MS, there is a need to understand the extent to which oculomotor disorders confound visual cognitive assessments. Therefore, the aim of this study was to determine whether, and to what extent, disrupted eye movements due to INO affect performance on the SDMT and other visual cognitive tests, as compared to cognitive tests with auditory stimuli. We hypothesised that INO may affect visual cognitive test performance as a tests reliance on rapid saccades and INO severity increases, while test with auditory stimuli should not be affected.

## Methods

### Trial design and participants

This retrospective analysis included 197 PwMS with oculography data available from the single-centre, prospective, observational PrograMS cohort study. As previously described, participants were between 18 and 80 years old, were diagnosed with clinically definite MS based on the 2017 revised McDonald criteria, and their disease course was categorised as relapsing–remitting, secondary progressive, or primary progressive MS [13, 25, 33, 38, 45]. Data collection took place between July 2015 and February 2018 for baseline, and between May 2021 and December 2023 for follow-up.

### Data acquisition

Participants were invited for a baseline and follow-up visit that consisted of an interview, clinical assessment, neuropsychological assessment, magnetic resonance imaging (MRI) scan, and infrared oculography.

### History and clinical assessment

Demographic and MS-related information was collected for all participants. Disease duration was calculated from the year of the first manifestation of neurological symptoms suggestive of MS. Visual acuity was assessed using ETDRS charts at 2 m, with refractive correction using participants’ glasses or contact lenses as required. Disability was evaluated using the Expanded Disability Status Scale (EDSS), Nine-Hole Peg Test (NHPT), and Timed 25-Foot Walk (T25FW).

### Neuropsychological assessment

PwMS were assessed with an expanded brief repeatable battery of neuropsychological tests, including tests with auditory or visual stimuli: the oral SDMT, the Paced Auditory Serial Addition Test 3-second version (PASAT-3), the Selective Reminding Test (SRT), the 10/36 Spatial Recall Test (SPART), the Word List Generation Test (WLG), the Stroop Colour Word Test (Stroop), the Memory Comparison Test (MCT), and the Concept Shifting Test (CST) [22, 29, 33, 37, 39]. Table 1 shows an overview of these cognitive tests and their main outcomes, main cognitive domain and cognitive abilities [31]. Analyses focussed on these main outcomes most often used to assess test-specific cognitive abilities, and other outcomes are reported as supplementary materials. Demographic-corrected cognitive test scores were calculated using regression-based age, sex, and education (years) norms from the healthy control (HC) dataset and converted to *Z*-scores using the mean and standard deviation from the HCs per visit.

**Table 1:** Components of the neuropsychological examination

Cognitive test	Test stimulus	Main outcome used	Main cognitive domain	Cognitive abilities
SDMT	Visual	Total score, 90 s trial	Complex attention	Information processing speed and sustained attention
PASAT-3	Auditory	Total score, 3 s trial	Complex attention	Information processing speed and sustained attention
SRT	Auditory	Total words in long-term storage (LTS)	Learning and memory	Verbal recent memory and free recall
SPART	Visual	Total correct responses	Learning and memory	Visual–spatial recent memory and free recall
WLG	Auditory	Total words produced	Language	Expressive language (fluency)
Stroop	Visual	Stroop interference score	Executive function	Overriding habits/inhibition
MCT	Visual	4-letter trial time^a^	Executive function	Working memory
CST	Visual	Trial C time^a^	Executive function	Mental flexibility and inhibition

^a^Corrected for basic motor speed

### Magnetic resonance imaging

MRI acquisition and MRI processing have been previously described [45]. In short, participants were scanned on a 3.0T Discovery MR750t MRI system (General Electric, Milwaukee, WI), with an eight-channel phased-array head coil. Among other sequences, the scan protocol included a three-dimensional T1-weighted fast spoiled gradient echo sequence (3D-T1w: repetition time 8.0 ms, echo time 3.0 ms, inversion time 450 ms, flip angle 12°, 1.0 × 0.9 × 0.9 mm^3^ in-plane resolution), and a three-dimensional T2-weighted fluid-attenuated inversion recovery sequence (FLAIR: repetition time 8000 ms, echo time 125 ms, inversion time 2350 ms, 1.2 × 1.0 × 1.0 mm^3^ in-plane resolution). MRI post-processing was performed using a structural processing pipeline available on GitHub [4]. In short, 3D-T1w scans were bias field corrected and skull stripped. These scans were lesion filled using FLAIR-derived white matter hyperintensity masks. These images were then used to obtain the total brain volume (TBV) and cortical grey matter volume using FreeSurfer (v7.3.2). The thalamic volume (ThalV) was segmented using FSL FIRST (v6.0.7.5) on the skull-stripped bias-field corrected 3D-T1w images. All volumetric values were proportionally normalised using the estimated total intracranial volume (eTIV) provided by FreeSurfer. Segmentations were manually adjusted when necessary.

### Infrared oculography

Eye movements were assessed with an EyeLink 1000 Plus eye tracker (SR Research, Ottawa, Canada) and the prosaccades task of the DEMoNS oculography protocol [24]. The presence and severity of INO was determined using the versional dysconjugacy index (VDI) of the area under the curve of the saccadic trajectory (VDI-AUC) and peak velocity corrected for saccadic amplitude (VDI-pV/Am) obtained from 15-degree prosaccades, as previously described [25]. Participants were classified as having INO when VDI-AUC exceeded 1.174 or VDI-pV/Am exceeded 1.180 on either leftward or rightward 15-degree saccades [25].

### Statistical analyses

Statistical analyses were performed in R (version 4.4.3). Statistical significance was set at *p*=0.05. Data was visually assessed for normality using histograms. Differences between PwMS with INO and PwMS without INO were analysed using the independent samples *t*-test for normally distributed continuous variables, the Wilcoxon rank sum test for non-normally distributed continuous variables, and the Fisher exact test for categorical variables. The effect of INO and VDI on cognitive test scores was assessed using three linear regression models: (1) a model with raw test scores as outcome; (2) a model with demographically corrected test *Z*-scores as outcome; and (3) a model with demographically corrected test *Z*-scores as outcome with additional correction for disease duration, EDSS, cortical grey matter volume and thalamus volume. The third model aimed to correct for real differences in cognitive ability between PwMS with and without INO by incorporating MRI measures that are known to correlate with cognition. INO presence, VDI-AUC and VDI-pV/Am for leftward and rightward saccades were assessed in separate models as predictors. Longitudinal effects of new-onset INO on changes in test *Z*-score were investigated in a similar manner. Corrections for multiplicity were made test-wise for each cognitive test using the false discovery rate method by Benjamini and Hochberg [7].

## Results

### Participant characteristics

Participant characteristics are shown in Table 2. PwMS had a mean age of 54 years, a mean disease duration of 22 years, and a median EDSS score of 3.5. Women comprised two-thirds of the cohort. Most patients had a relapsing–remitting disease course (66%).

**Table 2:** Participant characteristics of PwMS with and without INO

	Overall *N* = 197^a^	PwMS with INO *N* = 66^a^	PwMS without INO *N* = 131^a^	*p*-value^b^
**Demographics**				
Age (years)	54 ± 11	56 ± 10	53 ± 11	0.077
Sex, female	130 (66%)	35 (53%)	95 (73%)	0.010
Education (years)	15 (14, 16)	16 (14, 16)	15 (14, 16)	0.670
Dominant hand (right)	175 (89%)	59 (89%)	116 (89%)	>0.999
**Disease-related characteristics**				
Disease duration from onset (years)	22 ± 9	23 ± 9	21 ± 8	0.057
MS-subtype				0.021
RRMS	130 (66%)	35 (53%)	95 (73%)	
SPMS	50 (25%)	24 (36%)	26 (20%)	
PPMS	17 (8.6%)	7 (11%)	10 (7.6%)	
Current DMT use (yes, %)	58 (29%)	16 (24%)	42 (32%)	0.256
DMT type^c^				0.190
High efficacy DMT	5 (8.6%)	3 (19%)	2 (4.8%)	
Moderate efficacy DMT	18 (31%)	3 (19%)	15 (36%)	
Low efficacy DMT	33 (57%)	10 (63%)	23 (55%)	
Other	2 (3.4%)	0 (0%)	2 (4.8%)	
BCVA	1.00 (1.00, 1.25)	1.00 (1.00, 1.25)	1.25 (1.00, 1.25)	0.237
EDSS	3.5 (2.5, 6.0)	4.0 (3.0, 6.0)	3.5 (2.5, 4.5)	0.002
NHPT (sec)	20.58 (18.67, 24.53)	23.55 (20.40, 26.85)	19.75 (18.20, 22.44)	<0.001
T25FW (sec)	5.10 (4.20, 6.90)	5.80 (4.70, 7.80)	4.70 (4.00, 6.10)	<0.001
**Cognitive functioning**				
SDMT (*Z*-score)	−1.26 ± 1.06	−1.47 ± 1.11	−1.15 ± 1.02	0.051
PASAT-3 (*Z*-score)	−0.31 ± 1.42	−0.22 ± 1.38	−0.35 ± 1.44	0.571
CST-C (*Z*-score)^d^	−1.19 ± 2.38	−1.36 ± 1.81	−1.11 ± 2.62	0.441
SRT-LTS (*Z*-score)	−1.09 ± 1.58	−1.51 ± 1.77	−0.88 ± 1.44	0.014
WLG (*Z*-score)	−0.54 ± 0.90	−0.72 ± 0.89	−0.46 ± 0.89	0.058
SPART (*Z*-score)	−0.96 ± 1.32	−1.06 ± 1.31	−0.90 ± 1.33	0.420
MCT (*Z*-score)^d^	−0.39 ± 1.42	−0.40 ± 1.40	−0.39 ± 1.44	0.976
Stroop (*Z*-score)^d^	0.00 ± 1.46	−0.17 ± 1.69	0.09 ± 1.33	0.275
**MRI**				
nTBV (%)	70.6 ± 5.0	69.5 ± 5.9	71.1 ± 4.5	0.085
nCGMV (%)	31.50 ± 2.15	31.00 ± 2.56	31.75 ± 1.88	0.057
nThalV (%)	0.88 ± 0.12	0.85 ± 0.12	0.90 ± 0.12	0.027
**Oculography**				
Leftward VDI-AUC	1.078 (1.028, 1.140)	1.187 (1.105, 1.354)	1.065 (1.020, 1.093)	<0.001
Rightward VDI-AUC	1.083 (1.037, 1.142)	1.192 (1.090, 1.400)	1.054 (1.023, 1.104)	<0.001
Leftward VDI-pV/Am	1.069 (1.015, 1.137)	1.207 (1.088, 1.552)	1.049 (0.997, 1.090)	<0.001
Rightward VDI-pV/Am	1.066 (1.006, 1.124)	1.188 (1.092, 1.544)	1.036 (0.992, 1.082)	<0.001

^a^Mean ±SD; *n* (%); median (Q1, Q3)

^b^Welch two-sample *t*-test; Wilcoxon rank sum test; Fisher’s exact test

^c^Low efficacy DMT = Interferon-beta, glatiramer acetate and teriflunomide; moderate efficacy DMT = dimethyl fumarate, fingolimod, ponesimod, and siponimod; high efficacy DMT = ocrelizumab, natalizumab, and ofatumumab

^d^*Z*-scores reversed so lower scores indicate worse performance

*PWMS* persons with multiple sclerosis, *INO* internuclear ophthalmoplegia, *RRMS* relapsing–remitting MS, *SPMS* secondary progressive MS, *PPMS* primary progressive MS, *DMT* disease-modifying therapy, *BCVA* best corrected visual acuity, *EDSS* Expanded disability status scale, *T25FW* Timed 25-Foot Walk test, *NHPT* Nine hole peg test, *SDMT* Symbol digit modalities test, *PASAT*-*3* Paced auditory serial addition test (3 seconds trial), *CST*-*C* Concept shifting test (trial C), *SRT*-*LTS* Selective reminding test (long term storage), *WLG* Word list generation, *SPART* 10/36 spatial recall test total, *MCT* Memory comparison test (4 letter trial), *Stroop* Stroop colour word test (interference score), *nTBV* normalised total brain volume, *nCGMV* normalised cortical grey matter volume, *nThalV* normalised thalamus volume, *VDI* versional dysconjugacy index, *AUC* area under the curve, *pV*/*Am* peak velocity divided by amplitude

Sixty-six PwMS were diagnosed with INO: 21 with INO on leftward gaze, 19 with INO on rightward gaze, and 26 with bilateral INO. Compared to PwMS without INO, PwMS with INO were more likely to be male (47% vs. 27%, *p*=0.010), more often had progressive disease (47% vs. 28%, *p*=0.021), had greater disability (EDSS median 4.0 vs. 3.5, *p*=0.002), worse arm function (NHPT 23.55 vs. 19.75 s, *p*<0.001), worse mobility (T25FW 5.80 vs. 4.70 s, *p*<0.001), worse verbal memory (SRT-LTS *Z*-score −1.51 vs. −0.88, *p*=0.014) and lower thalamic volume (nThalV 0.85% vs. 0.90% *p*=0.027).

INOs detected by the VDI-AUC criterion had a median VDI of 1.344 (range 1.175–1.935), and INOs detected using the VDI-pV/Am criterion had a median VDI of 1.359 (range 1.181–3.003).

### Effects of INO presence on cognitive test performance

Table 3 shows the effect of INO presence on cognitive test performance, regardless of the severity of INO. The presence of INO alone is not significantly associated with differences in performance on visual cognitive tests after correction for disability, disease duration, cortical grey matter volume, and thalamus volume.

**Table 3:** The effect of INO presence (yes/no) in relation to the main outcomes considered for visual and auditory cognitive tests

	*Z*-score^a^	Std. β	*p*-value
**Visual tests**			
SDMT (score, 90 s trial)	−0.11	−0.10	0.501
CST-C (trial C time)^b^	−0.37	−0.23	0.181
SPART (total correct responses)	0.27	0.21	0.201
MCT (time 4-letter trial)^b^	0.18	0.13	0.457
Stroop (interference score)	−0.24	−0.17	0.318
**Auditory tests**			
PASAT-3 (score, 3 s trial)	0.25	0.20	0.257
SRT-LTS (total words long-term storage)	−0.57	−0.36	0.022
WLG (total words)	−0.20	−0.22	0.190

Raw cognitive test scores are corrected for age, gender, and education level, and converted to *Z*-scores based on healthy control data. Linear regression models are adjusted for disease duration, EDSS, cortical grey matter volume, and thalamus volume

^a^Lower *Z*-scores indicate worse performance

^b^Trial time corrected for basic motor speed

*std*. *β* standardised beta coefficient, *SDMT* Symbol digit modalities test, *CST*-*C* Concept shifting test (trial C), *SPART* 10/36 spatial recall test, *MCT* Memory comparison test (4 letter trial), *Stroop* Stroop colour word test, *PASAT*-*3* Paced auditory serial addition test (3 second trial), *SRT*-*LTS* Selective reminding test (long term storage), *WLG* Word list generation

PwMS with INO had significantly lower raw SDMT scores (mean reduction −4; 95%CI −7 to −1) compared to PWMS without INO. However, after correcting for disease characteristics, there was no significant difference in SDMT performance between groups (*p*=0.501). The PASAT, which measures overlapping cognitive abilities but depends on auditory rather than visual processing, was not significantly affected by the presence of INO.

The presence of INO was not significantly associated with the main outcome of the CST (trial C). However, PwMS with INO performed significantly worse on the less complex trial B (ascending letters, lower cognitive load) of the CST (*Z*-score −0.83, 95%CI −1.49 to −0.18) even after correction for disease characteristics (Table S1).

PwMS with INO performed significantly worse on the main outcome considered for the Selective Reminding Test, even after correction for disease characteristics (SRT-LTS *Z*-score −0.57; 95%CI −1.07 to −0.08). INO was also associated with reduced performance on other SRT outcomes, including consistent long-term storage and delayed recall (Table S1).

The four-letter trial of the MCT, which has the highest cognitive demand and is the main outcome considered for the MCT, was not affected by INO. However, on the percentage sign and one-letter trials with low cognitive demand, PwMS with INO performed worse than PwMS without INO (MCT percentage trial *Z*-score −0.77; 95%CI −1.47 to −0.07). This effect diminished with increasing task complexity, as the relative demand on oculomotor function decreased (Table S1).

### Effects of INO severity and horizontal dysconjugacy on cognitive test performance

To assess whether more severe INO could affect cognitive test performance, the effect of horizontal dysconjugacy (VDI) on cognitive testing was analysed. Table 4 reports the effects of VDI-AUC and VDI-pV/Am for leftward and rightward gaze.

**Table 4:** The effect of horizontal dysconjugacy (VDI) on main outcomes of cognitive test performance

		Leftward gaze			Rightward gaze		
	VDI type	*Z*-score^a^	Std. β	*p*-value	*Z*-score^a^	Std. β	*p*-value
**Visual**							
SDMT (score, 90 s trial)	VDI-AUC	−0.93	−0.13	0.068	−0.39	−0.06	0.384
	VDI-pV/Am	−0.83	−0.24	<0.001^b^	−0.34	−0.10	0.168
CST-C (trial C time)^c^	VDI-AUC	−2.00	−0.18	0.019^b^	−0.13	−0.01	0.856
	VDI-pV/Am	−1.45	−0.27	<0.001^b^	−0.65	−0.12	0.135
SPART (total correct responses)	VDI-AUC	0.56	0.06	0.398	0.68	0.09	0.236
	VDI-pV/Am	0.00	0.00	0.997	0.35	0.08	0.273
MCT (time 4-letter trial)^c^	VDI-AUC	−0.15	−0.02	0.844	−0.64	−0.08	0.320
	VDI-pV/Am	−0.03	−0.01	0.936	−0.09	−0.02	0.798
Stroop (interference score)	VDI-AUC	−0.84	−0.09	0.271	0.12	0.01	0.854
	VDI-pV/Am	−0.50	−0.11	0.179	−0.20	−0.04	0.579
**Auditory**							
PASAT-3 (score, 3 s trial)	VDI-AUC	0.33	0.04	0.651	−0.34	−0.04	0.582
	VDI-pV/Am	0.32	0.06	0.465	−0.16	−0.04	0.647
SRT-LTS (total words long-term storage)	VDI-AUC	−1.30	−0.12	0.098	−0.80	−0.09	0.239
	VDI-pV/Am	−0.81	−0.15	0.037	−0.40	−0.08	0.288
WLG (total words)	VDI-AUC	−0.04	−0.01	0.930	−0.78	−0.15	0.058
	VDI-pV/Am	−0.23	−0.07	0.335	−0.38	−0.13	0.095

Raw cognitive test scores are corrected for age, gender, and education level and converted to Z-scores based on healthy control data. Linear regression models are adjusted for disease duration, EDSS, cortical grey matter volume, and thalamus volume

^a^Lower *Z*-scores indicate worse performance

^b^Bold values significant after correction for multiple testing (FDR-method)

^c^Trial time corrected for basic motor speed

*std*. *β* standardised beta coefficient, *VDI* versional dysconjugacy index, *AUC* area under the curve, *pV*/*Am* peak velocity divided by amplitude, *SDMT* Symbol digit modalities test, *CST*-*C* Concept shifting test (trial C), *SPART* 10/36 spatial recall test, *MCT* Memory comparison test, *Stroop* Stroop colour word test, *PASAT*-*3* Paced auditory serial addition test (3 s trial), *SRT*-*LTS* Selective reminding test (long term storage), *WLG* Word list generation

**Fig. 1:**
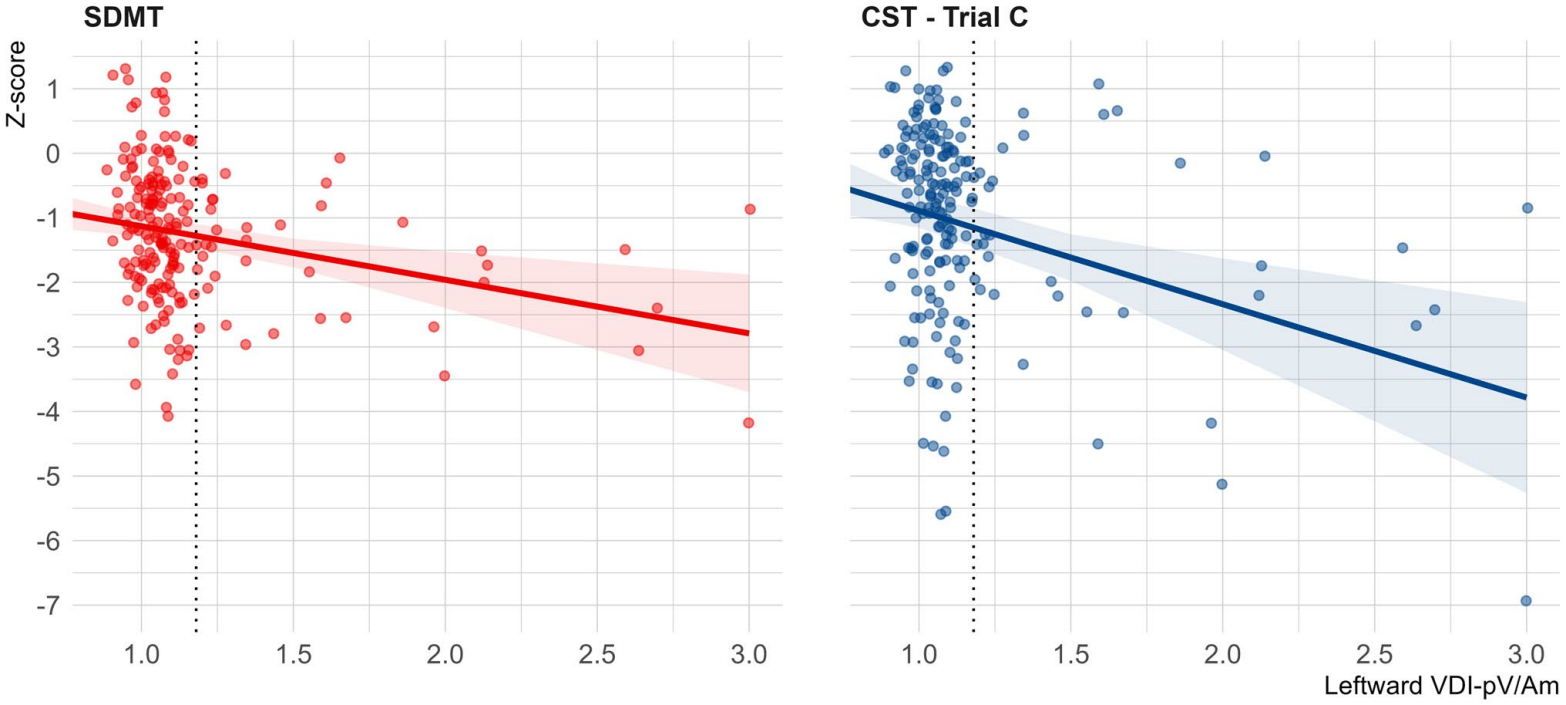
Effect of leftward versional dysconjugacy on the SDMT and CST. Data points show SDMT and CST *Z*-scores of PwMS, corrected for age, sex, and education. Regression lines show the predicted demographically corrected *Z*-score additionally corrected for disease duration, EDSS, cortical grey matter, and thalamus volume. The shaded area represents the 95% confidence interval of the regression line. The vertical dotted line represents the cutoff value for INO. *SDMT* Symbol digit modalities test, *CST* Concept shifting test, *VDI* versional dysconjugacy index, *pV*/*Am* peak velocity divided by saccadic amplitude

As dysconjugacy on leftward gaze measured by VDI-pV/Am increases, SDMT performance decreases. Specifically, every 0.1 increase in leftward VDI-pV/Am was associated with a −0.08 (95% CI −0.13 to −0.03) decrease in SDMT *Z*-score. Within the same statistical model, an increase in EDSS of one point resulted in a similar decline in SDMT *Z*-score (−0.12, 95%CI −0.20 to −0.03). Performance on the CST was associated with leftward VDI-pV/Am and VDI-AUC, where every 0.1 increase in leftward VDI was associated with a −0.14 (95% CI −0.22 to −0.07) and −0.20 (95% CI −0.37 to −0.03) decrease in CST-C *Z*-score, respectively. Figure 1 visualises the estimated effect of leftward dysconjugacy on SDMT and CST performance. VDI of rightward gaze affects SDMT performance after correction for demographics, but not after correction for disease characteristics. CST training trials A (ascending numbers) and B (ascending letters) were also associated with leftward VDI (*p*<0.001) (Table S2).

Auditory tests, such as the PASAT, were generally not associated with VDI, with the exception of the SRT, where consistent long-term storage (*Z*-score −0.79; 95% CI −1.37 to −0.22) and delayed recall (*Z*-score −1.27; 95% CI −2.06 to −0.48) were associated with leftward VDI-pV/Am (Table S2).

### Longitudinal follow-up

Follow-up was available for 102 PwMS with a mean follow-up time of 6.2 ±0.9 years. PwMS lost to follow-up (48%) more often had INO at baseline (42% vs. 25%), were older (57 vs. 51 years), had longer disease duration (23 vs. 20 years), more often had progressive disease (48% vs. 22%), were more disabled (median EDSS 4.0 vs. 3.5), had lower brain volume (nTBV 69% vs. 72%), and performed worse on the SDMT (*Z*-score −1.5 vs. −1.0), PASAT (*Z*-score −0.66 vs. 0.01), WLG (*Z*-score −0.76 vs. −0.35) and SPART (*Z*-score −1.21 vs. −0.73).

Among PwMS with follow-up available, new-onset INO was identified in seven participants (6.9%). Without correction for changes in disease characteristics, PwMS with new-onset INO showed a greater decline in CST-C performance (*Z*-score −1.87, 95% CI −3.04 to −0.71) than participants who did not develop INO. In the small sample of two participants with new-onset INO on leftward gaze, this effect was greater (*Z*-score −3.96, 95% CI −5.88 to −2.05).

Leftward VDI-pV/Am remained mostly stable over time, with considerable variation among PwMS (median −0.011; IQR −0.033, 0.035). With increasing leftward VDI-pV/Am, there was a trend for a decrease in CST-C performance (trial time +22.43s, 95% CI +2.55 to +42.31; demographically corrected *Z*-score −2.43, 95% CI −4.90 to +0.04). With increasing rightward VDI-AUC, there was a trend for a decrease in CST-C performance (trial time +16.18, 95%CI +0.60 to +31.76; demographically corrected *Z*-score −1.86, 95% CI −3.79 to +0.07) and SDMT performance (Score −10.32, 95% CI −19.37 to −1.28; demographically corrected *Z*-score −0.51, 95% CI −1.38 to +0.37). Neither trend was significant after adjustment for demographics. Performance on auditory tests was not associated with changes in VDI

## Discussion

Our results demonstrate that severe INO, particularly during leftward gaze, can impair performance on visual cognitive tests such as the SDMT and CST, independent of true underlying cognitive ability. While sufficient visual acuity is generally recognised as necessary for tests such as the SDMT, the potential impact of eye movement disorders is rarely considered, despite these assessments relying heavily on rapid saccades [34]. Our findings indicate that only more pronounced dysconjugacy, rather than the mere presence of mild INO, substantially distorts visual cognitive test results. This suggests that dysconjugacy exists on a continuum in PwMS, where most PwMS with mild INO may not experience functional visual impairments or can compensate for them, but as dysconjugacy increases, adaptation becomes insufficient and test performance is affected. This is especially relevant when clinical examination detects INO, as such cases are likely to be more severe [17].

Specifically, we found that a 0.1 increase in leftward VDI-pV/Am corresponded to a 0.08 decrease in SDMT *Z*-score. To illustrate, two individuals with MS with similar disability, disease duration and brain atrophy, one without dysconjugacy and the other with a right-sided INO with leftward VDI-pV/Am of 1.452 (95% CI 1.285–2.090), would be expected to differ by approximately four points on the SDMT solely due to INO. A four-point difference on the SDMT is traditional considered as a clinically important difference, although higher thresholds have been proposed [36, 43]. A right-sided INO (on leftward gaze) of this severity was found in 9.1% (95% CI 4.1–11.7%) of participants. Moreover, the negative impact on CST performance was even more pronounced. Given that leftward VDI-pV/Am ranged up to 3.0, INO may substantially distort visual cognitive test performance in some severe cases.

We found the effect of INO on visual cognitive tests was strongest with leftward gaze, possibly reflecting the left-to-right reading direction typically used in many cognitive tests, where a leftward ‘return-sweep’ saccade is required to find the new line [19]. While reading fatigue is frequently reported by patients with INO, this is, to our knowledge, the first study to suggest that left- versus right-sided INO may have differing effects on reading [16, 28]. Alternatively, right-sided brainstem lesions may have specific cognitive effects. Brainstem lesions have been found to be associated with deficits in executive functioning and attention, although the effect of lesion lateralisation on cognition needs to be explored [12].

Importantly, INO did not affect the PASAT, which relies on auditory stimuli to assess similar cognitive abilities as the SDMT. This suggests that using auditory tests may be a viable alternative for patients with severe INO [2, 44]. Additionally, we observed that INO had a greater effect on the training trials of tests such as the CST and MCT compared to more complex trials. This suggests that increasing task complexity and cognitive load may mitigate the impact of INO on cognitive test performance by reducing relative oculomotor demand.

Unexpectedly, INO was associated with poorer performance on the SRT for verbal memory. Although a mechanistic link between INO and verbal memory is not immediately apparent, there is evidence that eye movements contribute to verbal memory [30]. The proposed mechanism is that during the encoding of verbal information eye movements are made that provide a spatial component to the memory that is encoded along with the verbal information. During memory retrieval this spatial component elicits an eye movement to and fixation on the region in space that represented the verbal information during encoding, also termed the “looking at nothing” (LAN) phenomenon. LAN behavior was found to promote retrieval of auditorily encoded information, while memory retrieval was worse when eye movements were experimentally directed away from the area associated with to-be-retrieved information [32]. Eye movement disorders, like INO, may disrupt LAN behaviour and consequently impair verbal memory. Future studies that combine detailed eye tracking with verbal memory tasks are required to determine whether eye movement disorders indeed impair LAN behaviour and whether such changes mediate verbal memory impairment.

There are several limitations to this study that should be acknowledged. First, eye movement disorders other than INO were not assessed. Although INO is the most common eye movement disorder in MS, other abnormalities can occur and may affect cognitive test performance. These include horizontal or vertical gaze palsies due to brainstem or cranial nerve lesions, saccadic dysmetria, and various forms of nystagmus, particularly pendular nystagmus [23]. Unlike for INO, a similarly well-validated oculography-based framework to diagnose and quantify the severity of these other eye movement disorders was not available in this study. Residual confounding by eye movement disorders other than INO therefore cannot be excluded. Second, to account for potential confounding between INO and underlying cognitive ability, analyses were adjusted for demographics, disability, disease duration, cortical grey matter volume, and thalamic volume, assuming that participants with similar values should exhibit comparable cognitive ability. Nevertheless, we acknowledge that residual confounding may remain. Finally, our ability to longitudinally confirm the cross-sectional findings is limited by the small number of participants with new-onset INO at follow-up and by loss to follow-up among more affected, disabled, or older participants, introducing attrition bias [45]. Participants lost to follow-up may have been more likely to develop new-onset INO or to experience greater cognitive deterioration. The effect of new-onset INO on cognitive test performance may therefore be an underestimation, although the direction of the association is likely robust.

To gain further insight into how eye movement disorders impact visual cognitive tests, future research should investigate the effects of other, less common eye movement disorders, such as pendular nystagmus. Furthermore, alternative tests for cognitive processing speed may be developed that depend less on horizontal saccades, for example, by increasing the use of vertical saccades or minimising the need for eye movements altogether. A smartphone-based or computerised SDMT may be less dependent on horizontal saccades due to the smaller screen size, which reduced the required saccade amplitude, and the ability to present symbols sequentially in the centre of the screen, thereby reducing the necessity for substantial eye movements as required in the paper version [1, 20, 40]. We hypothesise that the impact of INO is smaller for a smartphone-based SDMT, but this needs to be confirmed by future studies. Moving beyond MS, future studies should investigate whether eye movement disorders affect visual cognitive test performance in other neurological diseases that can affect the brainstem, such as stroke, malignancies, and neurodegenerative disorders like progressive supranuclear palsy [11, 42]. Eye movement disorders may confound the measurement of cognitive ability in these diseases, making it difficult to accurately assess the efficacy of treatments focusing on cognitive improvement or prevention of disease progression.

Our results indicate that severe INO, particularly during leftward gaze, can impair performance on visual cognitive tests such as the SDMT and CST, independent of true cognitive ability. We recommend caution when interpreting these assessments in patients with clinically detectable INO. To mitigate the potential confounding effects of oculomotor dysfunction, clinicians may consider: (1) using a broader range of tests rather than relying on single visual tests, such as the SDMT, to assess cognitive function; (2) reducing the importance of eye movements by increasing task complexity and cognitive load; and (3) minimising the need for eye movements altogether, for example, through the use of digital or auditory cognitive tests. Ultimately, tailored assessment strategies that account for eye movement abnormalities may improve the accuracy of cognitive evaluation.

## Supplementary Information

Below is the link to the electronic supplementary material.

Supplementary file1 (PDF 531 KB)

## Data Availability

Data are available upon reasonable request.
