# Supplementary material for: What if it is the eyes? The effect of eye movement disorders on cognitive testing in MS
Source: J Neurol. 2026 Jun 2;273(6):355. doi: 10.1007/s00415-026-13905-y (PMC13230272; doi:10.1007/s00415-026-13905-y)
Supplement: Supplementary file 1 — Supplementary file1 (PDF 531 KB) [file 415_2026_13905_MOESM1_ESM.pdf]

Supplementary Information

"What if it's the eyes? The effect of eye-movement disorders on cognitive testing in MS"
Journal of Neurology

Authors:
S.N. Hof[1,2], E. Keytsman[3], T.A.N. Fuchs[2,4], M.H.J. Wessels[1,2], D.J. de Jong[1,2,4], E.M.M. Strijbis[1,2], M.M. Schoonheim[2,4], L.J. van Rijn[5,6], A. Petzold[7], B.M.J. Uitdehaag[1,2], J. Van Schependom[3,8], B.W. van Oosten[1,2]

[1] MS Center and Neuro-ophthalmology Expertise Center Amsterdam, Amsterdam UMC location Vrije Universiteit Amsterdam, Neurology, De Boelelaan 1117, Amsterdam, The Netherlands.
[2] Amsterdam Neuroscience, Neuroinfection & -inflammation, Amsterdam, The Netherlands
[3] AIMS Laboratory, Centre for Neurosciences, Vrije Universiteit Brussel, 1050 Brussels, Belgium
[4] MS Center Amsterdam, Amsterdam UMC location Vrije Universiteit Amsterdam, Anatomy & Neurosciences, De Boelelaan 1117, Amsterdam, The Netherlands.
[5] Neuro-ophthalmology Expertise Center Amsterdam, Amsterdam UMC location Vrije Universiteit Amsterdam, Ophthalmology, De Boelelaan 1117, Amsterdam, The Netherlands.
[6] Onze Lieve Vrouwe Gasthuis, Ophthalmology, Amsterdam, The Netherlands.
[7] The National Hospital for Neurology and Neurosurgery, Moorfields Eye Hospital and the Queen Square Institute of Neurology, UCL, London, United Kingdom.
[8] Department of Electronics and Informatics (ETRO), Vrije Universiteit Brussel, 1050 Brussels, Belgium.

Corresponding author: S.N. Hof, s.n.hof@amsterdamumc.nl

Table S1: The effect of INO in relation to cognitive test performance 2
Table S2: The effect of VDI in relation to cognitive test performance 3

Table S1: The effect of INO in relation to cognitive test performance

	Raw test scores			Corrected for demographics[1]			Corrected for demographics and MS[2]		
	Score	Std. β	p-value[3]	Z-score[4]	Std. β	p-value[3]	Z-score[4]	Std. β	p-value[3]
**SDMT**									
Score, 90s trial	-3.98	-0.38	**0.013**	-0.32	-0.30	**0.044**	-0.11	-0.10	0.501
**PASAT**									
Score, 3s trial	0.88	0.09	0.563	0.13	0.09	0.577	0.25	0.20	0.257
**CST**									
Trial A time, corrected for basic motor speed	2.76	0.41	**0.007**	-0.49	-0.31	0.049	-0.41	-0.25	0.147
Trial B time, corrected for basic motor speed	4.31	0.49	**0.001**	-0.95	-0.46	**0.003**	-0.83	-0.40	**0.013**
Trial C time, corrected for basic motor speed	2.45	0.14	0.371	-0.25	-0.11	0.495	-0.37	-0.23	0.181
**SRT**									
Total words long term storage	-6.67	-0.42	**0.005**	-0.63	-0.40	**0.008**	-0.57	-0.36	0.022
Total words consistent long term storage	-6.94	-0.41	**0.006**	-0.38	-0.31	**0.039**	-0.31	-0.25	0.108
Total words delayed recall	-1.15	-0.45	**0.003**	-0.73	-0.42	**0.005**	-0.55	-0.32	0.038
**WLG**									
Total words	-2.01	-0.29	0.057	-0.26	-0.29	0.057	-0.20	-0.22	0.190
**SPRT**									
Total correct responses	-0.64	-0.13	0.407	-0.16	-0.12	0.422	0.27	0.21	0.201
Total correct responses delayed recall	-0.18	-0.08	0.588	-0.03	-0.02	0.897	0.36	0.25	0.121
**MCT**									
Time percent sign trial	5.08	0.55	**<0.001**	-1.60	-0.55	**<0.001**	-0.77	-0.33	0.032
Time 1 letter trial	3.71	0.37	**0.014**	-0.84	-0.36	**0.018**	-0.57	-0.25	0.141
Time 2 letter trial	3.49	0.21	0.170	-0.41	-0.20	0.201	-0.12	-0.07	0.658
Time 3 letter trial	4.09	0.25	0.102	-0.38	-0.23	0.135	-0.03	-0.02	0.919
Time 4 letter trial	3.82	0.18	0.229	---	---	---	---	---	---
Time 4 letter trial, corrected for basic motor speed	0.07	0.00	0.977	-0.01	0.00	0.976	0.18	0.13	0.457
**Stroop**									
Word trial time	3.55	0.31	0.043	-0.62	-0.30	0.049	-0.37	-0.19	0.265
Colour trial time	4.31	0.30	0.045	-0.50	-0.28	0.068	-0.32	-0.18	0.274
Interference trial time	10.92	0.35	0.022	-0.56	-0.28	0.067	-0.54	-0.31	0.074
Stroop interference score	5.88	0.18	0.237	-0.26	-0.18	0.236	-0.24	-0.17	0.318

[1]Raw test scores corrected for age, gender and education level and converted to Z-scores based on healthy control data.[2]Raw test scores corrected for age, gender and education level, converted to Z-scores based on healthy control data and corrected for disease duration, EDSS, cortical grey matter volume and thalamus volume. [3]Bold values significant after test-wise correction for multiple testing (FDR method). [4] Lower Z-scores indicate worse performance. Abbreviations: std. β = standardized beta coefficient, SDMT = Symbol Digit Modalities Test, CST = Concept Shifting Test, SPART = 10/36 Spatial Recall Test, MCT = Memory Comparison Test, Stroop = Stroop colour-word test, PASAT = Paced Auditory Serial Addition Test, SRT = Selective Reminding Test, WLG = Word List Generation.

Table S2: The effect of VDI in relation to cognitive test performance

	Direction Gaze	VDI type	Raw test scores			Corrected for demographics[1]			Corrected for demographics and MS[2]		
			Score	Std.β	p-value[3]	Z-score[4]	Std. β	p-value[3]	Z-score[4]	Std. β	p-value[3]
**SDMT**											
Score, 90s trial	Left	AUC	-12.13	-0.17	**0.019**	-1.40	-0.19	**0.007**	-0.93	-0.13	0.068
		pV/Am	-8.01	-0.26	**<0.001**	-0.86	-0.27	**<0.001**	-0.83	-0.24	**<0.001**
	Right	AUC	-10.10	-0.17	**0.020**	-0.95	-0.16	**0.029**	-0.39	-0.06	0.384
		pV/Am	-5.02	-0.15	**0.033**	-0.58	-0.18	**0.014**	-0.34	-0.10	0.168
**PASAT**											
Score, 3s trial	Left	AUC	-0.22	0.00	0.966	-0.04	0.00	0.961	0.33	0.04	0.651
		pV/Am	2.27	0.07	0.349	0.34	0.07	0.346	0.32	0.06	0.465
	Right	AUC	-2.98	-0.05	0.481	-0.44	-0.05	0.484	-0.34	-0.04	0.582
		pV/Am	0.87	0.03	0.708	0.13	0.03	0.705	-0.16	-0.04	0.647
**CST**											
Trial A time, corrected for basic motor speed	Left	AUC	8.61	0.18	**0.011**	-2.05	-0.19	**0.011**	-1.61	-0.14	0.068
		pV/Am	5.14	0.26	**<0.001**	-1.17	-0.25	**<0.001**	-1.44	-0.27	**<0.001**
	Right	AUC	5.99	0.15	**0.032**	-1.24	-0.14	0.061	-1.01	-0.11	0.173
		pV/Am	3.19	0.14	0.045	-0.71	-0.14	0.059	-0.65	-0.12	0.138
Trial B time, corrected for basic motor speed	Left	AUC	17.06	0.28	**<0.001**	-3.92	-0.27	**<0.001**	-3.61	-0.25	**<0.001**
		pV/Am	8.83	0.34	**<0.001**	-2.00	-0.33	**<0.001**	-2.51	-0.36	**<0.001**
	Right	AUC	7.81	0.15	**0.033**	-1.79	-0.15	0.036	-1.34	-0.11	0.135
		pV/Am	4.70	0.16	**0.024**	-1.06	-0.16	0.030	-0.97	-0.14	0.066
Trial C time, corrected for basic motor speed	Left	AUC	16.00	0.13	0.072	-2.28	-0.14	0.056	-2.00	-0.18	**0.019**
		pV/Am	8.68	0.17	**0.020**	-1.21	-0.17	**0.016**	-1.45	-0.27	**<0.001**
	Right	AUC	4.17	0.04	0.573	-0.45	-0.03	0.652	-0.13	-0.01	0.856
		pV/Am	5.08	0.09	0.227	-0.71	-0.09	0.208	-0.65	-0.12	0.135
**SRT**											
Total words long term storage	Left	AUC	-10.17	-0.09	0.190	-1.15	-0.11	0.138	-1.30	-0.12	0.098
		pV/Am	-5.96	-0.13	0.075	-0.63	-0.14	0.060	-0.81	-0.15	0.037
	Right	AUC	-14.24	-0.16	0.028	-1.42	-0.16	0.029	-0.80	-0.09	0.239
		pV/Am	-5.10	-0.10	0.148	-0.59	-0.12	0.096	-0.40	-0.08	0.288
Total words consistent long term storage	Left	AUC	-15.56	-0.14	0.058	-1.07	-0.13	0.075	-1.08	-0.13	0.070
		pV/Am	-8.84	-0.18	**0.012**	-0.63	-0.18	**0.015**	-0.79	-0.20	**0.007**
	Right	AUC	-22.24	-0.23	**0.001**	-1.40	-0.20	**0.005**	-0.92	-0.13	0.074
		pV/Am	-7.21	-0.14	0.054	-0.48	-0.13	0.076	-0.39	-0.10	0.167
Total words delayed recall	Left	AUC	-2.71	-0.15	0.031	-1.96	-0.17	**0.020**	-1.87	-0.16	0.024
		pV/Am	-1.68	-0.22	**0.002**	-1.16	-0.23	**0.001**	-1.27	-0.22	**0.002**
	Right	AUC	-3.18	-0.22	**0.003**	-2.11	-0.21	**0.003**	-1.41	-0.14	0.050
		pV/Am	-0.90	-0.11	0.116	-0.69	-0.13	0.075	-0.34	-0.06	0.399
**WLG**											
Total words	Left	AUC	-1.04	-0.02	0.762	-0.12	-0.02	0.788	-0.04	-0.01	0.930
		pV/Am	-1.54	-0.08	0.295	-0.20	-0.07	0.306	-0.23	-0.07	0.335
	Right	AUC	-7.23	-0.18	**0.011**	-0.94	-0.18	**0.011**	-0.78	-0.15	0.058
		pV/Am	-2.72	-0.13	0.079	-0.37	-0.13	0.062	-0.38	-0.13	0.095
**SPRT**											
Total correct responses	Left	AUC	-0.74	-0.02	0.768	-0.17	-0.02	0.793	0.56	0.06	0.398
		pV/Am	-2.10	-0.14	0.051	-0.44	-0.11	0.117	0.00	0.00	0.997
	Right	AUC	-2.11	-0.07	0.314	-0.40	-0.05	0.462	0.68	0.09	0.236
		pV/Am	-0.71	-0.04	0.535	-0.13	-0.03	0.666	0.35	0.08	0.273
Total correct responses delayed recall	Left	AUC	-0.29	-0.02	0.788	-0.25	-0.03	0.712	-0.03	0.00	0.963
		pV/Am	-0.34	-0.05	0.474	-0.21	-0.05	0.467	-0.21	-0.04	0.559
	Right	AUC	-0.40	-0.03	0.665	-0.13	-0.02	0.818	0.79	0.10	0.207
		pV/Am	-0.24	-0.04	0.624	-0.15	-0.03	0.633	0.31	0.07	0.367

Table S2: The effect of VDI in relation to cognitive test performance

	Direction Gaze	VDI type	Raw test scores			Corrected for demographics[1]			Corrected for demographics and MS[2]		
			Score	Std.β	p-value[3]	Z-score[4]	Std. β	p-value[3]	Z-score[4]	Std. β	p-value[3]
**MCT**											
Time percent sign trial	Left	AUC	8.65	0.14	0.054	-2.58	-0.13	0.071	-1.93	-0.13	0.082
		pV/Am	4.47	0.17	0.021	-1.35	-0.16	0.028	-1.36	-0.18	0.013
	Right	AUC	9.92	0.19	0.008	-3.13	-0.19	0.009	-1.45	-0.11	0.132
		pV/Am	5.35	0.19	0.008	-2.09	-0.22	**0.002**	-1.19	-0.15	0.040
Time 1 letter trial	Left	AUC	8.70	0.13	0.074	-2.24	-0.14	0.049	-1.44	-0.09	0.228
		pV/Am	4.96	0.17	0.018	-1.16	-0.17	0.018	-1.17	-0.15	0.048
	Right	AUC	5.54	0.10	0.178	-1.42	-0.11	0.141	-0.43	-0.03	0.682
		pV/Am	4.00	0.13	0.072	-1.32	-0.17	0.017	-0.54	-0.07	0.388
Time 2 letter trial	Left	AUC	9.61	0.08	0.242	-1.10	-0.08	0.284	-0.48	-0.04	0.558
		pV/Am	5.50	0.11	0.121	-0.65	-0.11	0.145	-0.50	-0.10	0.213
	Right	AUC	6.55	0.07	0.344	-0.79	-0.07	0.364	0.20	0.02	0.776
		pV/Am	4.91	0.09	0.189	-0.78	-0.11	0.117	-0.03	-0.01	0.944
Time 3 letter trial	Left	AUC	12.99	0.12	0.108	-1.23	-0.11	0.130	-0.56	-0.05	0.486
		pV/Am	8.68	0.18	0.013	-0.84	-0.17	0.016	-0.67	-0.13	0.091
	Right	AUC	12.12	0.13	0.074	-1.18	-0.13	0.083	-0.29	-0.03	0.678
		pV/Am	7.24	0.14	0.049	-0.86	-0.16	0.028	-0.18	-0.03	0.663
Time 4 letter trial	Left	AUC	19.03	0.13	0.063	---	---	---	---	---	---
		pV/Am	9.50	0.15	0.031	---	---	---	---	---	---
	Right	AUC	16.18	0.14	0.059	---	---	---	---	---	---
		pV/Am	7.74	0.12	0.096	---	---	---	---	---	---
Time 4 letter trial, corrected for basic motor speed	Left	AUC	10.54	0.09	0.190	-0.92	-0.09	0.189	-0.15	-0.02	0.844
		pV/Am	4.57	0.10	0.187	-0.40	-0.10	0.186	-0.03	-0.01	0.936
	Right	AUC	10.38	0.11	0.123	-0.90	-0.11	0.123	-0.64	-0.08	0.320
		pV/Am	3.64	0.07	0.319	-0.32	-0.07	0.321	-0.09	-0.02	0.798
**Stroop**											
Word trial time	Left	AUC	6.62	0.08	0.246	-1.06	-0.08	0.299	-0.69	-0.05	0.497
		pV/Am	3.71	0.11	0.132	-0.61	-0.10	0.162	-0.72	-0.11	0.153
	Right	AUC	9.64	0.14	0.044	-1.71	-0.15	0.045	-0.79	-0.07	0.368
		pV/Am	4.59	0.13	0.077	-1.07	-0.16	0.029	-0.47	-0.07	0.378
Colour trial time	Left	AUC	13.09	0.13	0.061	-1.50	-0.12	0.091	-1.05	-0.09	0.244
		pV/Am	6.96	0.16	0.021	-0.82	-0.16	0.032	-0.95	-0.17	0.032
	Right	AUC	12.07	0.15	0.040	-1.47	-0.14	0.049	-0.85	-0.09	0.279
		pV/Am	5.84	0.13	0.067	-0.86	-0.15	0.044	-0.39	-0.06	0.414
Interference trial time	Left	AUC	35.33	0.16	0.022	-2.27	-0.17	0.020	-2.15	-0.18	0.021
		pV/Am	16.90	0.18	0.011	-1.05	-0.18	0.012	-1.35	-0.24	**0.003**
	Right	AUC	22.48	0.12	0.083	-1.24	-0.11	0.131	-0.72	-0.07	0.377
		pV/Am	11.24	0.12	0.109	-0.76	-0.12	0.106	-0.50	-0.08	0.308
Stroop interference score	Left	AUC	19.33	0.09	0.229	-0.87	-0.09	0.228	-0.84	-0.09	0.271
		pV/Am	9.50	0.10	0.170	-0.43	-0.10	0.169	-0.50	-0.11	0.179
	Right	AUC	3.20	0.02	0.813	-0.14	-0.02	0.811	0.12	0.01	0.854
		pV/Am	3.25	0.03	0.656	-0.15	-0.03	0.655	-0.20	-0.04	0.579

[1]Raw test scores corrected for age, gender and education level and converted to Z-scores based on healthy control data. [2]Raw test scores corrected for age, gender and education level, converted to Z-scores based on healthy control data and corrected for disease duration, EDSS, cortical grey matter volume and thalamus volume. [3]Bold values significant after test-wise correction for multiple testing (FDR method). [4] Lower Z-scores indicate worse performance. Abbreviations: VDI = Versional Dysconjugacy Index, AUC = Area under saccadic Curve, pV/Am = peak velocity divided by saccadic amplitude, std. β = standardized beta coefficient, SDMT = Symbol Digit Modalities Test, CST = Concept Shifting Test, SPART = 10/36 Spatial Recall Test, MCT = Memory Comparison Test, Stroop = Stroop colour-word test, PASAT = Paced Auditory Serial Addition Test, SRT = Selective Reminding Test, WLG = Word List Generation.
